# Deciphering the roles of triiodothyronine (T3) and thyroid-stimulating hormone (TSH) on cardiac electrical remodeling in clinical and experimental hypothyroidism

**DOI:** 10.1007/s13105-023-01000-z

**Published:** 2023-11-29

**Authors:** Oscar Casis, Leire Echeazarra, Beatriz Sáenz-Díez, Mónica Gallego

**Affiliations:** Department of Physiology, Faculty of Pharmacy, University of the Basque Country UPV/EHU, Paseo de la Universidad 7, 01006 Vitoria-Gasteiz, Spain

**Keywords:** Electrical remodeling, Arrhythmia, Cardiac electrophysiology, Endocrine disease

## Abstract

Hypothyroidism is the most frequent endocrine pathology. Although clinical or overt hypothyroidism has been traditionally associated to low T3 / T4 and high thyrotropin (TSH) circulating levels, other forms exist such as subclinical hypothyroidism, characterized by normal blood T3 / T4 and high TSH. In its different forms is estimated to affect approximately 10% of the population, especially women, in a 5:1 ratio with respect to men. Among its consequences are alterations in cardiac electrical activity, especially in the repolarization phase, which is accompanied by an increased susceptibility to cardiac arrhythmias. Although these alterations have traditionally been attributed to thyroid hormone deficiency, recent studies, both clinical trials and experimental models, demonstrate a fundamental role of TSH in cardiac electrical remodeling. Thus, both metabolic thyroid hormones and TSH regulate cardiac ion channel expression in many and varied ways. This means that the different combinations of hormones that predominate in different types of hypothyroidism (overt, subclinic, primary, central) can generate different forms of cardiac electrical remodeling. These new findings are raising the relevant question of whether serum TSH reference ranges should be redefined.

## Introduction

Although not as famous as diabetes, hypothyroidism (HT) is actually the most prevalent endocrine disease in Western countries. In Spain, almost half of the endocrinology patients are treated for thyroid diseases [23]. Primary hypothyroidism, the most frequent thyroid disease, is characterized by a decrease in circulating levels of the thyroid hormones thyroxine (T3) and triiodothyronine (T4). This is caused by a deficient function of the thyroid gland, accompanied by a compensatory elevation of pituitary thyroid-stimulating hormone (thyrotropin or TSH) (Table 1). Its prevalence is higher in women, with a female-to-male ratio of 5:1 in European countries [6, 23, 37]. The prevalence of HT in industrialized countries is not very different: around 0.6% in Europe, 0.35% in the USA, and 0.7% in Korea [22, 32, 39].

**Table 1:** Different types of hypothyroidism are characterized by different levels of circulating hormones

	TSH	T3
Overt HT	↑↑↑	↓↓
Subclinical HT	↑	=
Central	↓↓↓	↓↓

Subclinical hypothyroidism is a silent form of HT in which the thyroid gland is damaged, but normal T3 and T4 levels are maintained at the expense of a compensatory elevation of TSH levels. Since blood T3 levels are normal, most patients remain undiagnosed. According to the Spanish Society of Endocrinology and Nutrition [9], only 600,000 people are diagnosed with subclinical hypothyroidism (1.3% of the population), but the estimated amount could be close to 3 million patients (around 6%) [11]. In Europe, the prevalence is around 4.5%; in the USA, between 4.3 and 8.5%; and in Korea 3.1% [22, 32, 39]. Very importantly, patients with subclinical HT have an estimated probability of progressing to clinical HT between 5–20% at 1 year, and 63% at 10 years. In any case, this progression seems to be proportional to elevated TSH levels, advanced age, and the presence of antithyroid antibodies [5, 21, 27].

Finally, central hypothyroidism is an infrequent form of HT caused by insufficient stimulation of the thyroid gland due to low levels of thyroid-stimulating hormone (Table 1). Central HT can be secondary, whose origin is pituitary, or tertiary, with hypothalamic origin. In children, it is usually caused by craniopharyngiomas and anterior cranial irradiation of brain tumors or hematologic malignancies. In adults, it is usually due to macroadenomas, pituitary surgery, or post-irradiation. The estimated prevalence of central HT is 1/80,000 to 1/120,000 worldwide [24].

One of the main targets of thyroid hormones is the cardiovascular system [30]. The main cardiac symptoms of HT are poor exercise tolerance and increased fatigue [13]. In more advanced stages, ventricular and atrial fibrosis, myocardial edema, and reduced cardiac output usually appear [10, 50, 58]. The effects of thyroid dysfunction on cardiac and cardiovascular mechanical function, such as heart failure and coronary disease, have recently been reviewed [67]. However, it should be noted that the first signs of cardiac dysfunction are often found on the electrocardiogram. It is known that cardiac arrhythmias can be elicited or maintained by circulatory factor such as inflammatory cytokines or by structural remodeling such as myocardial infarction or interstitial fibrosis, and their involvement in arrhythmogenesis in hypothyroidism has also been recently reviewed [57]. Therefore, through a historical perspective, this review aims to delve specifically into the causes of the cardiac electrical remodeling that occurs in patients with hypothyroidism and in experimental models of this disease.

## Cardiac electrical remodeling in hypothyroidism

### Primary overt hypothyroidism

**Fig. 1:**
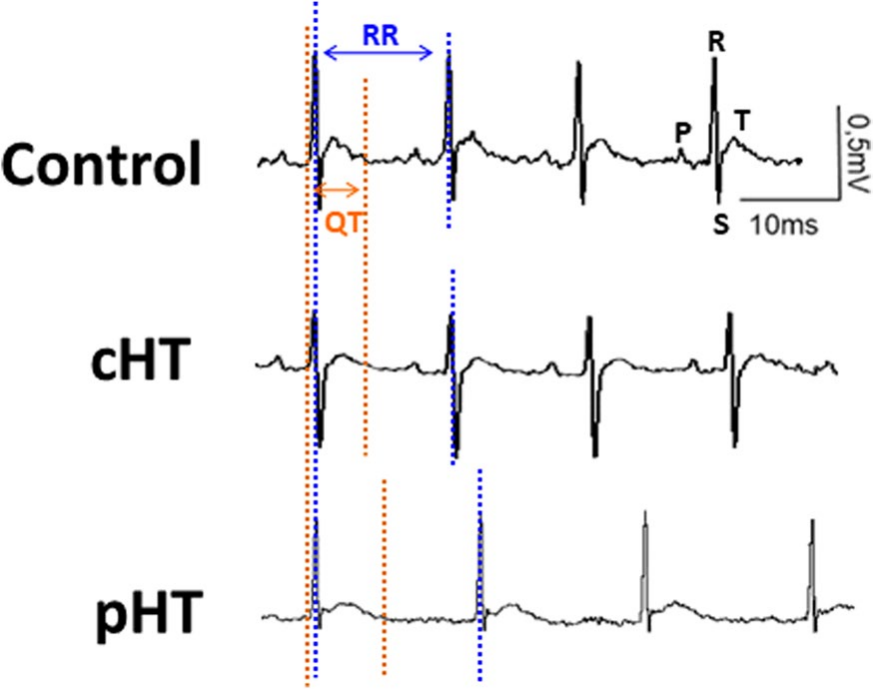
Original electrocardiographic recordings in experimental animals. Healthy (control), central (cHT), and primary hypothyroidism (pHT). The recording of the animal with central HT is very similar to the control recording, while that of the animal with primary HT presents severe alterations, such as atrial fibrillation and a lengthened QT interval. The recordings correspond to lead II. P, R, S, and T waves are indicated in the control recording. The Q wave is not visible in any recording. RR, RR interval; QT, QT interval

The electrocardiograms of hypothyroid patients often show significant sinus bradycardia, atrial fibrillation, and, mainly, prolonged ventricular repolarization (Fig. 1). Thus, there is a lengthening of the QT interval (indicator of the duration of ventricular depolarization), and of the rate-corrected QT (QTc). In addition, HT increases QT dispersion or QTd (which is the difference between the longest and shortest QT in all leads), and causes a prolongation of the duration of the T-peak to T-end (T_peak_–T_end_), reflecting an increase in ventricular electrical heterogeneity. Either QTc prolongation, QTd increase, or T_peak_–T_end_ lengthening is independently associated with an elevated incidence of polymorphic ventricular tachyarrhythmias, such as *torsades de pointes* (TdP). Although TdP usually extinguish spontaneously, if they persist, they can ultimately lead to ventricular fibrillation and sudden cardiac death [3, 4, 10, 20, 60]. Animal models of HT consistently reproduce the electrocardiographic alterations observed in patients [15, 16, 69].

### Subclinical hypothyroidism

Individuals affected by subclinical HT are usually asymptomatic but show electrocardiographic abnormalities similar to those seen in patients with overt hypothyroidism. Evidence of this is that the patients who have overcome Hasimoto’s thyroiditis and maintain normal T3/T4 levels at the expense of an elevated TSH have prolonged QTc and increased QTd. These patients, like those with overt HT, show a higher incidence of atrial and ventricular premature beats [21].

### Central hypothyroidism

Because the prevalence of central hypothyroidism is very low and patients usually have other hormonal deficits, the effects on the ECG have been poorly studied. However, experiments in animal models reveal that central HT does not cause significant electrocardiographic alterations (Fig. 1) [15].

The distinctive feature of central HT, what makes it different from primary clinical and subclinical HT, is that low levels of T3 and T4 are accompanied by low levels of TSH.

## Role of TSH in cellular cardiac electrical remodeling in hypothyroidism

Given that in subclinical HT thyroid hormone levels are still normal, the coincidence in electrocardiographic manifestations between established and subclinical HT suggests that cardiac electrical remodeling is not only due to thyroid hormone deficiency, but also to some other factor. In fact, incubation of human-induced stem cell-derived cardiac myocytes (hiPS-CMs) with low concentrations of T3 (0.01 nM) and T4 (0.0002 and 0.02 nM) did not cause lengthening of the field potential duration (FPD), which is the in vitro equivalent of the QT interval [59].

It is important to remember that overt and subclinical HT have in common high TSH levels. In this regard, several groups have described that in patients with HT, QTc lengthening and QTd increase reverse after treatment, when TSH levels normalize. Therefore, based on correlation analyses performed in healthy individuals and in hypothyroid patients, with or without treatment, several groups proposed that QTc and QTd alterations are caused by elevated TSH levels [21, 38, 60]. In the same line of research, a correlation between TSH concentration and the duration of the T_peak_–T_end_ has recently been described [4].

Obviously, for this to be true, the TSH receptor must be expressed in cardiac muscle. In the last decades, TSH receptor expression has been described in several extrathyroidal tissues such as skin, kidney, liver, bone, blood vessels, or the immune system, although its physiological relevance is difficult to establish and is still a matter of debate [62]. In the heart, the TSH receptor was first described in mouse atria and human cardiac muscle in 1995 [14]. Later studies in cardiac ventricles of rodents confirmed its expression at the mRNA and protein level and, more importantly, found that TSH receptors in the heart activate the cAMP and PKA pathway [12, 26]. More recently, experiments in isolated rat ventricular cardiomyocytes performed by our research group demonstrated that incubation with TSH regulates the expression of Kv4.3, Kv4.2, and Kir2.1 K^+^ channels and the accessory protein KCHIP2, whereas blockade of the TSH receptor with a specific antibody prevented the effect [2]. Similarly, in adult human atrial myocytes, PKA inhibition suppresses the TSH-induced effect [15]. This demonstrates that TSH regulates cardiac electrical activity directly, through activation of its receptor and its canonical signaling pathway.

## Hormones responsible for cellular cardiac electrical remodeling in hypothyroidism

In the human heart, the action potential (AP) is the result of a balance between depolarizing and repolarizing currents. The fast inward Na^+^ current (I_Na_), carried by Nav1.5 channels, which are codified by the *SCN5A* gene, depolarizes the phase 0 of the cardiac AP, whereas the inward L-type Ca^2+^ current (I_Ca-L_), carried by Cav1.2 channels, which are the product of the *CACNA1C* gene, depolarizes the plateau phase. On the other hand, the main four repolarizing K^+^ currents are as follows: the transient outward (I_to_), the rapid delayed rectifier (I_Kr_), the slow delayed rectifier (I_Ks_), and the ultrarapid delayed rectifier (I_Kur_). These currents are generated by the outflow of potassium through Kv4.3; Kv11.1 or hERG; Kv7.1 or KCNQ1, together with KCNE1; and Kv1.5 channels, which are codified by the *KCND3*, *KCNH2*, *KCNQ1/*KCNE1, and *KCNA5* genes, respectively. Therefore, the HT-induced lengthening of the ECG QT interval can be caused by an increase in depolarizing currents, a decrease in repolarizing currents, or a combination of both.

In the 1990s, the role of thyroid hormones in the regulation of cardiac ionic currents began to be studied intensively. However, the results obtained were in many cases contradictory, due to differences in the animal models and the techniques used between the different studies. After 30 years of research, the picture is beginning to become clearer.

### Na^+^ and Ca^2+^ currents

Patch-clamp experiments in hypothyroid guinea pigs showed that HT had no effect on I_Na_ [8]. This result was confirmed by microarray analysis in hypothyroid rats, where sodium channel subunit transcripts were unaffected [33].

Although that work in guinea pigs also found no effect on the I_Ca-L_ [8], subsequent research has consistently contradicted this result. Thyroid hormones regulate I_Ca-L_ at both the transcriptional and post-transcriptional levels. On the one hand, T3 acutely increases the calcium current by acting directly on rat cardiac myocytes and activating the cAMP cascade [64]. On the other hand, it inhibits the expression of L-type Ca^2+^ channels. Consequently, animals with primary or central hypothyroidism have higher expression of *CACNA1C* gene and higher I_Ca-L_ amplitude compared with euthyroid animals [15, 33, 64]. This increase in I_Ca-L_ may contribute to the lengthening of cardiac repolarization observed in hypothyroid patients. Last, regarding the role of TSH, our group has found no effect on I_Ca-L_ amplitude or *CACNA1C* channel expression [2, 15].

### Repolarizing K^+^ currents

#### Transient outward K^+^ current

Since I_to_ in rodents has a very large current amplitude, it has been extensively studied. Pioneering studies in neonatal cardiac myocytes demonstrated that thyroid hormones were necessary for correct transient outward current development, that is, for normal channel protein expression and normal current amplitude [52, 53, 65]. These findings led to several studies in rats with myocardial infarction who showed reduced I_to_, and found that treatment with thyroid hormones and hormone analogues like 3,5-diiodothyropropionic acid (DITPA) restored I_to_ density and expression of Kv4.2 and Kv1.4 channels [45, 66]. In addition, treatment with DITPA also restored cardiac I_to_ in diabetic animals with hypothyroidism [17]. In fact, HT per se, reduces the amplitude of several potassium currents, including I_to_. Our group found that the decrease in I_to_ amplitude is not homogeneous along the ventricular wall, which, in turn, explains the alterations in QT dispersion and T_peak_–T_end_ duration [16] observed in hypothyroid animals.

Interestingly, unlike its crucial role for normal I_to_ development, it has also been consistently demonstrated that T3 has no effect on this current in adult cardiac myocytes, either healthy or hypothyroid [53, 56]. Therefore, the absence of T3 does not explain the reduction of I_to_ observed in adult hypothyroidism.

Conversely, we found a direct relationship between TSH and reduced I_to_ in hypothyroidism [2, 15]. Both current amplitude and Kv4.3 channel expression were reduced in cardiac myocytes isolated from animals with primary HT (with high TSH), but not in those isolated from animals with central HT (with low TSH). We also observed that incubation with TSH reduced I_to_ in myocytes from control and central hypothyroid animals, but not in myocytes from animals with primary HT (in which the current was already reduced due to high plasma TSH levels). Furthermore, incubation with TSH reduced *KCND3* gene expression in human heart samples in vitro. Taken together, these results grant a novel role for TSH in cardiac electrical remodeling and support the hypothesis that QTc and QTd alterations are caused by elevated TSH levels.

#### Delayed rectifying currents I_Kr_, I_Ks_, and I_Kur_

In the human ventricle, the main repolarizing current is the rapid delayed rectifier or I_Kr_ that flows through the hERG channel. In human-induced stem cell-derived cardiac myocytes (hiPS-CMs), treatment with T3 stimulated myocytic maturation in terms of increased cell size, sarcomere length, and contractile force [68]. In a recent work in hIPS-CMs, combined T3+Dexamethasone treatment increased I_Kr_ expression and therefore improved the electrophysiological maturation of these cells [63]. However, neither hypo- nor hyperthyroidism modify the expression of the *KCNH2* gene, which encodes the I_Kr_ channels, in adult murine cardiomyocytes [33]. These results suggest that, as with I_to_, thyroid hormones are necessary for the normal development of I_Kr_ in neonatal myocytes, and that the effect is reduced throughout development until it disappears in adult cardiac myocytes. Finally, regarding the role of the thyroid-stimulating hormone, our group recently reported that incubation with TSH in human heart samples did not affect the expression of *KCNH2* gene [15].

The slow delayed rectifier K^+^ current, I_Ks_, is primarily responsible for the adaptation of repolarization duration to changes in heart rate and sympathetic stimulation. Again, T3+Dexamethasone-treated hIPS-CMs developed increased I_Ks_ expression [63], consistent with an important role for T3 in assisting electrophysiological maturation of cardiac cells. However, to our knowledge, the direct effect of thyroid hormones on I_Ks_ in adult cardiac myocytes has not been studied. In the context of hypothyroidism, the results on the I_Ks_ are contradictory. A first study reported a reduction of I_Ks_ amplitude in guinea pigs with primary hypothyroidism [65]. In contrast, later work in hypothyroid mice found that the expression of the I_Ks_-generating genes *KCNQ1* and *KCNE1* and the current amplitude were increased [33]. Further experiments would be needed to confirm the effect of thyroid hormones on adult cardiac myocytes, but in light of the results described above on I_Ca-L_, the guinea pig may not to be a good model for studying the electrophysiological effects of thyroid hormones [8, 15, 33, 64, 65]. On the other hand, we have observed that incubation with TSH reduces the expression of the *KCNQ1* gene in control adult human heart samples. This could explain, at least in part, the higher incidence of cardiac arrhythmias in hypothyroid patients under conditions of sympathetic stimulation, observed both in vivo and in silico [15].

Last, the ultrarapid outward current, I_Kur_, has little physiological relevance in the human ventricle but is essential for atrial repolarization. Both primary (with high TSH) and central HT (with low TSH) reduce Kv1.5 channel expression and I_Kur_ amplitude [15, 33, 47]. This finding, together with the absence of effect after incubation with TSH, indicates that I_Kur_ is directly regulated by T3 [2]. This could also explain the high incidence of atrial arrhythmias in hypothyroid patients.

## Cardiac electrical remodeling is different depending on the combination of hormones

**Fig. 2:**
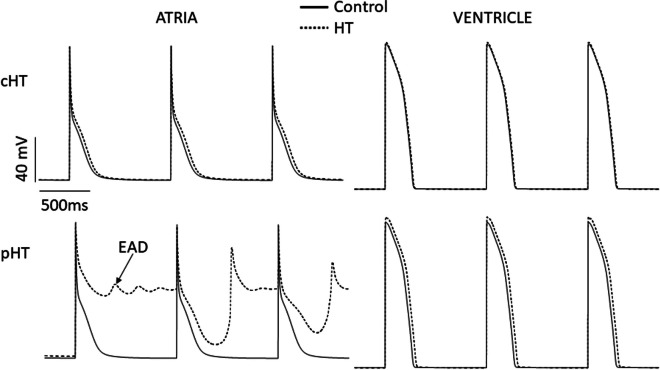
Human atrial and ventricular action potentials (AP) simulated in conditions of primary or central HT. Atrial and ventricular electrical remodeling caused by central hypothyroidism (cHT; low TSH) and primary hypothyroidism (pHT; high TSH). cHT significantly prolongs the duration of atrial AP, but only slightly that of ventricular AP. In pHT, repolarization in the atrium fails, which explains the appearance of atrial fibrillation on the ECG. Similarly, in pHT, the ventricular action potential also shows a significant alteration of repolarization, which explains the incidence of early afterpotentials (EAD) and extrasystoles. Atrial and ventricular action potentials were simulated using the Nygren-Firek-Clark-Lindblad-Clark-Giles and O’Hara-Rudy dynamic models, respectively

Since the expression of ionic currents is not homogeneous in the heart, action potential waveform is different in sinoatrial and atrioventricular nodes, atria, Purkinje fibers, and ventricular endocardium, midmyocardium, and epicardium. In addition, plasmatic levels of T3 and TSH are different in each type of HT, and these hormones selectively regulate ionic currents. Ultimately, several combinations of hormone levels and hormone-induced effects on almost all cardiac ionic currents have been extensively studied. These results can be introduced in mathematical models of atrial and ventricular action potentials [43, 44] to predict global effects in the heart (Fig. 2).

T3 hormone is necessary for the postnatal development of potassium currents such as I_to_, I_Kr_, and I_Ks_. Subsequently, the effect of this hormone on I_to_ and I_Kr_ currents is reduced throughout development and finally disappears in adult cardiomyocytes. In contrast, T3 hormone increases the expression of *CACNA1*, *KCNQ1*, and *KCNE1* genes and the amplitude of I_Ca-L_ and I_Ks_ currents in adult cardiac myocytes [33, 52, 53, 56, 63, 64, 68].

On the other hand, TSH has also direct effects on cardiac ion channel expression. Whereas TSH has no effect on the expression or amplitude of I_Ca-L_ or I_Kr_, it does reduce the expression and amplitude of I_to_, I_Ks_, and I_Kur_ through the activation of its receptor and the cAMP/PKA pathway [2, 15].

## Clinical implications

### Arrhythmogenic mechanisms

An accepted paradigm in cardiology is a substrate and a trigger are necessary for an arrhythmia to occur. Probably, the most common pro-arrhythmic substrate is QT prolongation [31, 41]. As explained, hypothyroidism modifies the functional expression of cardiac ionic currents, and this electrical remodeling often results in QT prolongation. Then, the main triggers of arrhythmia, which are early afterdepolarizations (EAD) and late afterdepolarizations (DAD), could act on this substrate.

DADs are often driven by spontaneous calcium release during diastole when intracellular Ca^2+^ overload increases the activity of the Na^+^/Ca^2+^ exchanger (NCX). Research from different groups demonstrated that, in hypothyroidism, the elevation of circulating TSH levels increases the expression of the NCX at the mRNA and protein level [15, 16, 33]. Surprisingly, although there are more NCX, its activity is inhibited by TSH [15]. In addition, hypothyroidism does not induce Ca^2+^ overload and even reduces Ca^2+^ transients and sarcoplasmic reticulum Ca^2+^ release [40]. Taken together, these results rule out DAD as the main trigger of arrhythmia in HT.

On the other side, in patients with prolonged QT interval, arrhythmias are often triggered by EADs [36]. EADs are voltage oscillations during the phase 2 or 3 of the action potential caused by the reactivation of depolarizing currents. The increase of I_Ca-L_ and the decrease of K^+^ currents prolong AP duration and can induce EADs due to the activation of the late sodium current, I_Na-L_ [25]. Our group has used the O’Hara-Rudy-dynamic model of ventricular AP [44] in in silico populations of control and hypothyroid patients and found higher incidence of EADs in the modeled hypothyroid patients [15]. In agreement with these simulations, a very recent paper demonstrated a high incidence of EADs in hypothyroid mouse hearts, which were abolished by the I_Na-L_-specific blocker ranolazine [54].

### Treatment of hypothyroidism

Hypothyroidism was treated with crude thyroid extract since the end of the nineteenth century. Later, a mixture of T3 and T4 began to be used, which continued to be used until the third quarter of the twentieth century. From then on, the use of T3 began to be eliminated because it was discovered that the organism transforms T4 into T3 and because exogenous T3 caused adverse effects. Although originally one of the reasons for the decline in the use of T3 in the treatment of HT was that it caused ventricular extrasystoles, it was demonstrated that treatment with either T4 or T3 improves cardiac electrical remodeling and reduces the incidence of arrhythmias in animal models of myocardial infarction and diabetes [17, 49, 65]. Currently, the standard treatment of hypothyroidism is daily levothyroxine (LT4) at the dose that normalizes TSH levels. However, it has been shown that this treatment does not eliminate symptoms in up to 20% of patients, and therefore, the introduction of T3 is being discussed again [9, 29, 51], although its effect on arrhythmogenesis in humans still needs to be studied in depth.

### Relevance of TSH levels

Serum TSH reflects thyroid status in a more sensitive way than free thyroxine, but it is only a valid measure when the hypothalamic-pituitary axis is intact. Although serum TSH measurement alone is not sufficient for the diagnosis of central hypothyroidism, currently, it is the best test to screen for primary hypothyroidism [48, 61].

The Framingham study [46] assessed whether TSH could affect left ventricular structure and function. No significant associations were observed between this hormone and left ventricular mass, wall thickness, or systolic function. However, TSH affects cardiac electrical behavior. In this sense, several clinical studies indicated that elevated serum TSH level played an essential role in QT prolongation and dispersion in patients with hypothyroidism [7, 21]. Experimental findings from our laboratory are consistent with this, since TSH directly modulates cardiac currents, prolongs repolarization, and increases the susceptibility to cardiac arrhythmias [2, 15].

A very recent study has revealed that hypothyroidism is an independent predictor of progression from paroxysmal AF to persistent AF after AF ablation, even after treatment and normalization of thyroxine levels. This supports the hypothesis that TSH could be involved in the remodeling, either electrical or structural, responsible for the appearance or maintenance of the AF substrate [35].

In addition, increased mortality and incidence of major adverse cardiovascular endpoints (MACE) have been reported among individuals with both high and low TSH levels, even within the range of values considered normal [1, 28, 42]. In the clinical setting, this raises the question of whether the reference ranges of serum TSH should be re-redefined [18, 19, 34, 55].

## Conclusion

Metabolic thyroid hormones are important in the development and maturation of potassium currents such as I_to_ and I_Kr_, but their effect on these currents vanishes during development. In adult cardiac myocytes, thyroid hormones modulate the expression and behavior of I_Ca-L_ and I_Ks_.

**Fig. 3:**
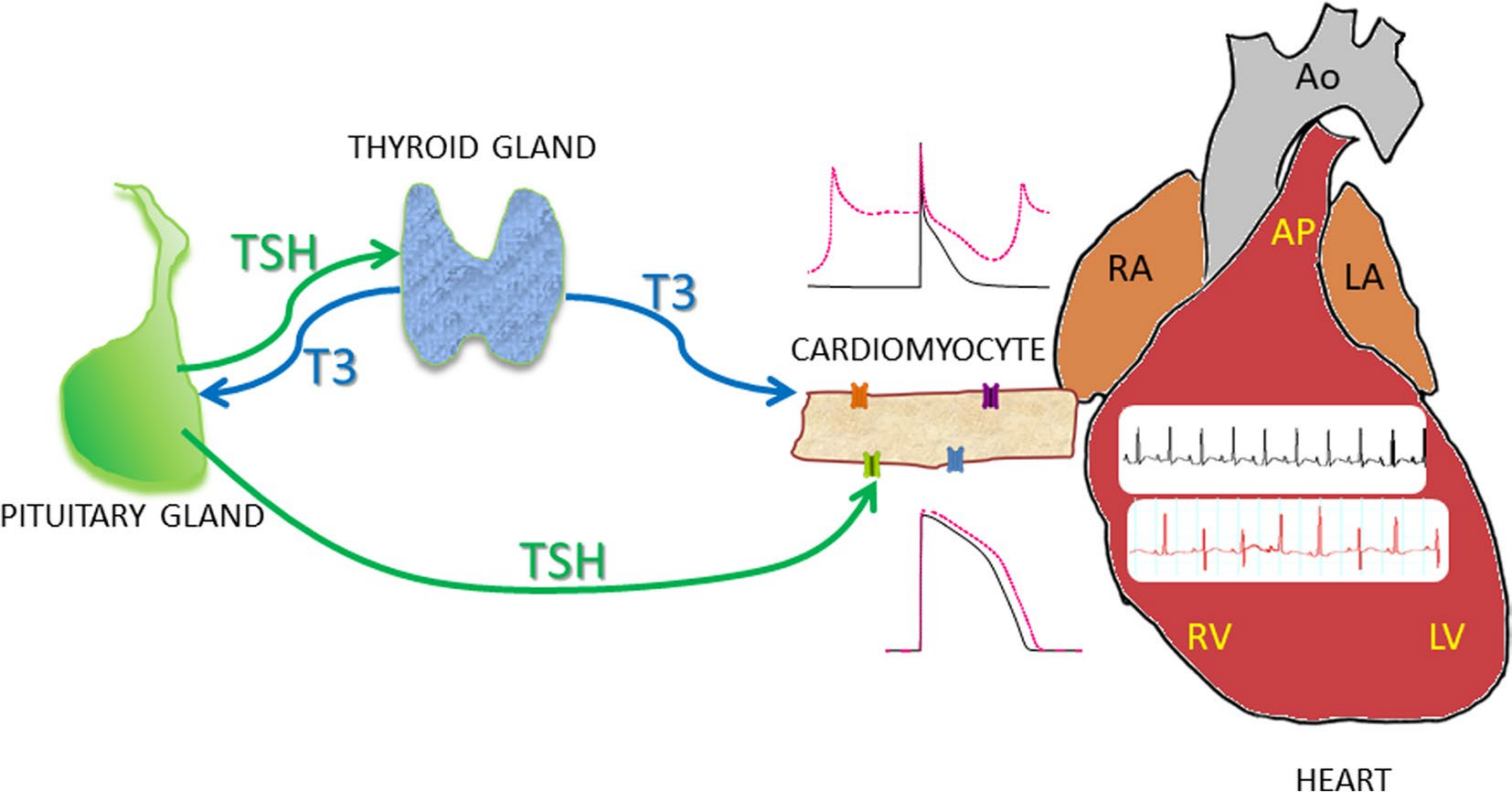
Both T3 and TSH are essential for the regulation of cardiac electrical activity. T3 regulates the expression of Ca^2+^ and K^+^ channels in the cardiac myocyte membrane in healthy conditions. TSH, in addition to modulating the release of T3 from the thyroid, has a direct effect on cardiac myocytes, as it regulates the expression of K^+^ channels. The balance between these hormones determines the shape and duration of the action potential in isolated cardiomyocytes, and the characteristics of the electrocardiogram of patients. Low T3 and high TSH increase Ca^2+^ channels and decrease K^+^ channel expression, and this deregulation leads to arrhythmia. (Healthy, black traces; HT, red traces; RA/LA, right/left atria; RV/LV, right/left ventricle; Ao/PA, aorta/pulmonar artery)

Thyroid-stimulating hormone has also important effects in the regulation of cardiac electrical activity. Through the activation of its receptor in the heart, TSH modulates the expression of the repolarizing currents I_to_, I_Ks_, and I_Kur_. The important role that this hormone plays in cardiac electrical remodeling and the appearance of cardiac arrhythmias that occur in hypothyroidism have recently been demonstrated (Fig. 3).
